# Validation of a family planning self-efficacy measure with married women in Bihar, India: Findings from the Bihar Integrated Family Planning Survey^☆^^☆☆^

**DOI:** 10.1016/j.conx.2024.100113

**Published:** 2024-11-13

**Authors:** Nandita Bhan, Edwin Elizabeth Thomas, Lotus McDougal, Priya Nanda, Tanmay Mahapatra, Aritra Das, Sweta Kumari, Kalysha Closson, Abhishek Singh, Anita Raj

**Affiliations:** a Jindal School of Public Health and Human Development, OP Jindal Global University, Sonipat, Haryana, India; b Center on Gender Equity and Health, University of California San Diego, La Jolla, CA, United States; c Independent Consultant; d Bihar Technical Support Program, Patna, India; e Department of Public Health and Mortality Studies, International Institute for Population Sciences, Mumbai, India; f Newcomb Institute of Tulane University, Tulane School of Public Health and Tropical Medicine, New Orleans, LA, USA

**Keywords:** Contraceptive autonomy, Demand-side determinants of family planning, Fertility intention, Reproductive agency, Reproductive autonomy, Self-efficacy

## Abstract

**Objectives:**

Adapting and testing a novel measure of family planning self-efficacy (FPSE) and examining its association with fertility intention and contraceptive use in India.

**Study design:**

Data were analyzed from 13,901 non-sterilized, currently married women of reproductive age (15-49 years) in the Bihar Integrated Family Planning Survey (BIFS) 2021. We adapted an FP Self Efficacy measure comprising women’s agency to overcome barriers to accessing, discussing and using contraception, regardless of family pressure and social judgment. We used factor analyses to assess reliability and validity, and regression analyses to examine the associations of FPSE with key family planning outcomes.

**Results:**

The study sample was relatively young (35% below 25 years of age), with 43% reporting no education and over half (52%) married before 18 years of age. The 9-item FPSE scale demonstrated high reliability (Cronbach’s α=0.82) with two factors – self-efficacy to access and discuss contraception versus self-efficacy to use contraception in the face of resistance. Higher FPSE was associated with spousal communication [AOR: 2.35 (95% CI: 2.18, 2.54), traditional [AOR: 1.24 (95% CI: 1.12,1.36)] and reversible modern contraception [AOR: 1.58 (95% CI: 1.43,1.75)], and fertility intention [AOR: 1.13 (95% CI: 1.01,1.25)].

**Conclusion:**

FP Self Efficacy was found to be a reliable and valid measure associated with spousal communication, reversible contraception use and fertility intention.

**Implications:**

Measures to capture reproductive agency, such as family planning self efficacy within FP programs, place women’s choice as central goals of FP programming and can help in meeting community needs and the demand for contraceptive use.

## 1 Introduction

The past decade has noted steady increases in contraceptive use in low-and-middle-income countries (LMICs), with sharp growth across sub-Saharan Africa and South Asia [1]. Increased access to contraceptives has been instrumental in averting more than 141 million unintended pregnancies, 29 million unsafe abortions and almost 150,000 maternal deaths [2], [3]. Despite these gains, a number of structural and social barriers continue to impede contraceptive access, especially among younger and low parity women, and these barriers exist irrespective of marital status [4]. These barriers relate not only to contraceptive availability and preparedness of health systems to meet increasing contraceptive demand, but also to gendered and social barriers to women’s choices for fertility, contraceptive use and family planning (FP). Previous work has only interrogated to a limited degree how women, irrespective of marital status negotiate these choices with their husbands or partners, and navigate them within their families and communities.

Within sexual and reproductive health (SRH) programs, while there is growing evidence on the association between women’s empowerment and contraceptive use [5], [6], [7], the pathways from empowerment to contraceptive use for women are not well understood. The translation of women’s empowerment and agency into measurable constructs and indicators useful for FP monitoring and evaluation programs remains a challenge. Reproductive agency of women is most often captured using the proxy measure of the household decision-making, which may not necessarily reflect the pathways to women’s family planning choice and use [8], [9].

In this context, the construct of self-efficacy may provide insight into women’s reproductive agency for contraceptive demand and use. Self-efficacy measures, capturing the “*belief in one’s capabilities to organize and execute the courses of action required to manage prospective situations*” [10] have been previously used in other contexts [11], [12] and may be situation specific or vary by the complexity of the ask, environment, contextual and social factors [13]. Women’s family planning self-efficacy (FPSE) captures the belief or confidence to plan, access, discuss, negotiate, and persist in the use of family planning. Family planning self efficacy considers not only the intrinsic capacity for FP discussion and use but also negotiations with external social forces (e.g. the views of partners and family members).

Most measures of self-efficacy in LMICs focus on contraceptive self-efficacy, and few offer reliability and validity statistics [14], [15], [16], [17], [18], [19], [20], [21], [22], [23], [24], [25], [26]. These measures have demonstrated associations with continued contraceptive use, [14], [16], [17], [18], [19], [20], [21], [22], [23], [24], [25], [26] increased use of prescription-based contraceptive methods [20] and oral contraceptive pill adherence [25]. Research from Kenya and Nigeria found contraceptive self-efficacy to be associated with the use of modern contraception at 12 months postpartum [26]. In the Democratic Republic of Congo (DRC), postpartum family planning self efficacy was associated with FP discussion with husband/partner or a health worker, as well as access and use of contraception [19]. Among men in Kenya, associations were noted between self-efficacy, intention to accept partner’s use of contraception, and masculine norms and FP acceptance [21]. Most measures of self-efficacy examine individual-level perceptions and few have explored the influence of parents, in-laws and peers that guide FP use. This influence can be important for women in considering, voicing, discussing and decision-making on family planning.

To this end, we adapted and tested a measure of FP Self Efficacy in India to capture reproductive agency and assess the predictive value of family planning self efficacy for key FP outcomes. Our findings provide insight on FP programming successes and gaps, and can contribute to designing more effective strategies for counseling and service delivery.

## 2 Materials and methods

### 2.1 Data and sample

We examined data from the Bihar Integrated Family Planning Survey (BIFS), a state-representative cross-sectional survey among currently married women of reproductive ages (15–49 years) in 2021. The BIFS survey was designed by CARE-India in partnership with the state government and development partners, and supported by members of the Family Planning Monitoring Learning and Evaluation Consortium (FP-MLE)^1^ and the Bill and Melinda Gates Foundation (BMGF). BIFS assessed intent, behaviors and practices related to FP as well as the reproductive profile, determinants of contraceptive use and uptake of FP services among 22,668 married women across all 38 districts in the state of Bihar, India. While Bihar has noted improvements in its contraceptive prevalence rate, modern spacing methods and a decline in total fertility rate, [27] the state notes high rates of unintended pregnancies compared to the national average [28]. In 2015, a report stated that 47.9% of pregnancies in the state were unintended, with the unintended pregnancy rate at 89 per 1000 women of reproductive age [28].

Sampling, Data Collection and Ethics: The cross-sectional study design followed a four-stage structure where primary sampling units were blocks (sampled from all 38 districts in Bihar to ensure geographical representativeness), secondary sampling units (SSU) were Anganwadi Centers (AWC) in rural areas and municipal wards in urban areas, and houses/ residential structures considered as the tertiary sampling units (TSU). The fourth/ final sampling stage was applicable when the selected house/structure had more than one eligible participant (currently married women aged 15–49 years). Interviews for the participants were conducted by female data collectors using structured interviews. The SSUs were sampled using simple random sampling from the line-list of AWCs/municipal wards and from each SSU, five TSUs were selected using systematic sampling with a random start and fixed interval. In the sampled TSUs with more than one eligible participant, one was sampled via random name-based sorting. The total planned sample size was 22,800 (600 women per district), of which interviews were successfully completed with 22,668 women (99.4%). Interviews with the participants were conducted by female data collectors using a pre-tested structured questionnaire (previously piloted in the same geography). The study was approved by the Ashirwad Ethics Committee, Ashirwad Hospital and Research Center, Ulhasnagar, India. Verbal informed consent was collected from each agreeing participant before the interview, after explaining the details of the study in Hindi. Further details on the survey design and ethics are available [29]. For this analysis, we restricted our sample to the 13,901 non-sterilized women.

### 2.2 Developing the family planning self-efficacy (FPSE) measure

FP Self Efficacy was first developed in Guatemala in 2016 as an 18 item measure to capture the barriers to realization of women’s reproductive agency and its measurement for FP programs and surveys [23], [30], [31]. FP Self Efficacy captured women’s self-efficacy to access contraception and contraceptive information, to communicate or assert contraceptive use in their relationship, and to use contraceptives despite social judgment or disapproval for such use, all of which were considered barriers to their agency for use. This conceptualization of FP Self Efficacy was contextualized within the framing of Can-Act-Resist [32] for this study. This framing captured dimensions such as consciousness or desire for FP, the choice or behavior that enabled “*can*” (desire for use) and “*act*” (actual use) for women, as well as the capacity or confidence to overcome barriers through “resist” [32]. Context-relevance of these items was assessed in an ongoing study in Uttar Pradesh, a neighboring state to Bihar, to understand item relevance, participant understanding and response patterns. Items that were redundant, or lacked salience in the Indian cultural context (“*family planning as a sin*”), were removed prior to pre-testing. We pre-tested using 10 cognitive interviews in Bihar to understand content validity and translation quality, clarity and comprehension of the questionnaire items. The interview findings were used to make decisions on retaining/deleting certain items and to improve translation. Items related to side-effects (e.g. continue to use family planning even in the context of fear of side effects) were removed as these were dependent on the quality of care related to information available on method-specific issues and side effects; similarly, items on convincing one’s partner to use male methods (male sterilization and use of condoms), considered a major barrier to use, were added. The 13-item FP Self Efficacy in the BIFS included self-efficacy to access contraceptive knowledge or contraceptives, discussion of FP methods with the husband, friends and family, using contraception in the face of opposition from husband or family, convincing husband to use a male-focused contraception, and contraceptive use in the face of community criticism.

### 2.3 Measures of family planning and sociodemographic factors

The survey assessed spousal communication by asking women if they had ever discussed FP with their husbands including ideal number of children, delaying first child, spacing between children or contraceptive use. Responses were coded as no (reference) vs yes.

The survey asked women if they had ever used contraception. Options offered included intra-uterine contraceptive devices (IUCD), injectables, oral contraceptive pills (OCPs), weekly OCPs/Centchroman, emergency contraceptive pills (ECPs), condoms, standard days method (SDM), lactational amenorrhea method (LAM), rhythm method, withdrawal and other traditional methods. Responses for *contraception ever use* were coded as no (reference) vs any contraception used.

We also assessed *contraceptive use by type*, categorized into no use, traditional methods (including withdrawal, rhythm method, standard days method and lactational amenorrhea method) and reversible modern contraceptive methods (including intrauterine devices, injectables, oral contraceptive pills, weekly contraceptive pills and emergency contraceptive pills, and condoms). We also examined *specific contraceptive use types* and present the results for IUCDs, condoms and pills, the predominant spacing contraceptives used in Bihar.

Additionally, we examined *fertility intention* among participants based on their last or current pregnancy. Women were asked if they had intended to become pregnant (current/last) or had wished to wait or had not wanted any or more children. Responses were combined for pregnant and non-pregnant women and coded as ‘‘wanted to get pregnant’’, ‘‘wanted to wait’’ and ‘‘did not want more’’.

Finally, we also assessed *current use* (none vs any) among women reported Yes when asked if they had ever used contraception. Hence this comprised a sub-sample compared to other family planning outcomes.

Key covariates included age, education, caste/tribe status, religion, family type, parity and age at marriage. Women’s education was categorized as no schooling, <5 years, 5–9 years, 10–12 years and 13+ years. Wealth index was estimated based on an aggregated weighted score of 28 different household items and the type of dwelling. A relative weight, corresponding to the median depreciated value was assigned to each item. The aggregate score was log-transformed and the participating families were categorized as per tertile distribution based on this score as high, medium and low wealth [33]. Caste-tribe status was categorized as scheduled castes (SC), scheduled tribes (ST), other backward classes (OBC) and general categories, and religion as Hindu vs Non-Hindu. These caste categorizations originate in article 366 of the Indian Constitution and other Constitutional panels and bodies, with the aim of legislative action, providing recognition to socioeconomically deprived communities and for affirmative action. Family type was categorized as nuclear vs non-nuclear, and parity was categorized as 0,1, 2, and 3 or more. Age at marriage was categorized as <18 years vs ≥18 years.

### 2.4 Analysis

We examined sociodemographic characteristics of the sample, and conducted exploratory factor analyses, using principal-component method, orthogonal varimax rotation, and iteratively building and testing models for 3 and 2 factor solutions and assessing model fit. Items were removed if factor loading cut-offs were less than 0.35, and if loadings were over 0.65, then considered for inclusion in the factor. The number of factors were included based on eigenvalues and using the elbow rule in the scree plot. Cronbach’s alpha was used to assess the internal consistency reliability, setting alpha = 0.7 as indicative of high reliability of the measure [34]. Inter-item correlations assessed clustering and cohesiveness of the sub-scales. A summative score was considered for the measure, dichotomized at the median into low vs high. We examined correlations between family planning self efficacy and household decision-making, women’s mobility and final say on FP decision-making to test construct validity. Household-decision-making and women’s mobility are well considered measures and have been adapted from the Demographic and Health Surveys (DHS), and capture aspects of women’s empowerment that conceptually align with FP self efficacy. We also examined construct convergent validity of FP Self Efficacy through associations with spousal communication, contraceptive use outcomes, and fertility intention using binary and multinomial logistic regression analyses. These models adjusted for age, education, caste/tribe status, religion, parity, age at marriage and family type.

## 3 Results

Our sample comprised 13,901 non-sterilized currently married women, who were relatively young (35% between 18–25 years of age) with limited education (43% had no schooling) (Table 1). A majority of women in the BIFS belonged to OBC castes (62%) and over 80% were Hindu. Three in five women (59.3%) reported living in a non-nuclear family, and over half (52%) had married prior to 18 years of age.

**Table 1:** Sociodemographic characteristics of the sample in the Bihar Integrated Family Planning Survey (BIFS) 2021 in India

Age (in years)	*N* = 13,901 (%)
<18 y	114 (0.8)
18−25	4898 (35.2)
25−35	5457 (39.7)
35−49	3432 (24.7)
*Years of schooling*	
No education	5988 (43.1)
<5 y	473 (3.4)
5−9 y	3304 (23.8)
10−12 y	2915 (20.9)
13+	1221 (8.8)
*Wealth index with 3 categories*	
Poor	4437 (31.9)
Middle	4648 (33.4)
Rich	4816 (34.6)
*Caste*	
Scheduled Castes (SC)	2618 (18.8)
Scheduled Tribes (ST)	118 (0.8)
Other Backward Classes (OBC)	8692 (62.5)
General	2473 (17.8)
*Religion*	
Hindu	11,582 (83.3)
Non-Hindu	2318 (16.7)
*Family type*	
Nuclear	5656 (40.7)
Non-nuclear	8245 (59.3)
*Parity (number of children)*	
0	1362 (9.8)
1	2871 (20.6)
2	3449 (24.8)
3+	6219 (44.7)
*Age at marriage (Child Marriage)*	
≥18 y (No)	6702 (48.2)
<18 y (Yes)	7199 (51.8)

While seven in 10 women in our study reported confidence to discuss ideal family size and FP methods with their husbands, most women did not report that same confidence in using an FP method without the knowledge or consent of their husbands (Table 2). Only one in three women (36.7%) felt confident that they could resist parental pressure, but community knowledge or criticism was not highlighted as a barrier to use.

**Table 2:** Original 13 family planning self-efficacy items [number (percent)] in the Bihar Integrated Family Planning Study (BIFS) 2021

Are you confident that you can	Not confident at all (0)	Somewhat confident (1)	Confident (2)
obtain information about different kinds of family planning methods.	2475 (17.8)	1954 (14.1)	9472 (68.1)
obtain a family planning method even if you have to wait in long lines.	3300 (23.7)	2208 (15.9)	8393 (60.3)
discuss how many children you want to have with your husband.	2187 (15.7)	1905 (13.7)	9809 (70.6)
discuss family planning methods with your husband.	2468 (17.7)	2089 (15.0)	9344 (67.2)
discuss family planning methods with your friends or relatives.	2871 (20.6)	2220 (15.9)	8810 (63.4)
use a family planning method even without discussing with your husband	9419 (67.8)	1621 (11.7)	2861 (20.6)
use a family planning method even if your husband does not want you to	9586 (68.9)	1692 (12.2)	2623 (18.9)
use a family planning method even if your mother-in law does not want you to.	7241 (52.1)	1978 (14.2)	4682 (33.7)
use a family planning method even if your parents do not want you to.	6729 (48.4)	2075 (14.4)	5097 (36.7)
convince your husband to use Condom.	7207 (51.8)	2070 (14.9)	4624 (33.3)
convince your husband to use Male sterilization.	8839 (63.6)	1726 (12.4)	3336 (24.0)
continue to use family planning method even if people in your community find out about your use	3284 (23.6)	2375 (17.1)	8242 (59.3)
use a family planning method even if your neighbors criticize you.	3055 (21.9)	2243 (16.1)	8603 (61.9)

Factor analyses of the original 13 items demonstrated a two factor solution (explaining 54% of the variation) and reliability α = 0.86 (0.79–0.87 across factors). (Table 3*, inter-item correlations in* Supplementary Table 1). Factor 1 comprised seven items and focused on accessing and discussing FP. Two items “*continue to use FP even if community finds out*” and “*use a FP method even if your neighbors criticize you*” showed factor loadings <0.35 and related to community barriers, and were removed. Six items loaded on to factor 2 and focused on use of FP in light of pressure. Two items on male-focused methods were removed due to poor factor loadings and the view that these comprised an important but specific component that did not align conceptually with other scale items.

**Table 3:** Factor analyses of the original 13-item and the revised 9-item family planning self-efficacy measures

	Original 13 item FP self efficacy measure (explained variance: 54.5%)		Revised 9 item FP self efficacy measure (explained variance: 64.6%)	
Are you confident that you can	Factor 1	Factor 2	Factor 1	Factor 2
obtain information about different kinds of family planning methods.	0.76		0.80	
obtain a family planning method even if you have to wait in long lines.	0.71		0.75	
discuss how many children you want to have with your husband.	0.79		0.83	
discuss family planning methods with your husband.	0.79		0.83	
discuss family planning methods with your friends or relatives.	0.71		0.73	
use a family planning method even without discussing with your husband		0.72		0.74
use a family planning method even if your husband does not want you to		0.81		0.83
use a family planning method even if your mother-in law does not want you to.		0.77		0.81
use a family planning method even if your parents do not want you to.		0.73		0.76
*convince your husband to use Condom.*		0.47		
*convince your husband to use Male sterilization.*		0.54		
*continue to use family planning method even if people in your community find out about your use*	0.66			
*use a family planning method even if your neighbors criticize you.*	0.68			
Cronbach's alpha	0.87	0.79	0.86	0.81
# of items	7	6	5	4
Average inter-item covariance	0.31	0.29	0.34	0.38

FP, family planning.

Items in italics were removed from the final version of the FP self efficacy measure.

Revised factor analyses of the 9-item FP Self Efficacy showed α = 0.82 (64% explained variation) (Table 3). Five factors loaded on to factor 1: SE to access and discuss FP (α = 0.86) and four items loaded on factor 2: SE to use contraception in the face of resistance (α = 0.81). The overall FP Self Efficacy summative score ranged from 9 to 27 (mean: 19.32, SD: 4.74), while factor 1 ranged from 5 to 15 [mean: 12.45 (SD: 3.10)] and factor 2 ranged from 4 to 12 [mean 6.87 (SD: 2.79)]. Different cut-offs were used for the overall and the sub-scale factors. Using median cut-offs, FP Self Efficacy was coded as low if the score was less than 19, and high if 19 and above. Factor 1 was coded as low (score < 11) and high (score ≥ 11) and factor 2 was coded as low (score < 9) and high (score ≥ 9). In our study, nearly 62% of women reported high FP Self Efficacy, nearly 75% reported high SE to discuss contraception but only 12% reported high SE to use contraception. SE to discuss and use FP were associated with each other (*p* < 0.0001). Only 14.9% of women who reported high SE to discuss contraception also reported SE to use contraception.

FP Self Efficacy was associated with age, education, caste/tribe, wealth, religion, family type, having two children, and age at marriage (Supplementary Table 2). We noted the same for factor 1, but not factor 2. FP Self Efficacy was associated with women’s household decision-making agency, women’s mobility and who has the final say on contraception (Supplementary Table 3). Bi-variate association of FP Self Efficacy demonstrated associations with spousal communication, contraceptive type, fertility intention but not current use of contraception (*p* = 0.1) (Table 4).

**Table 4:** Bi-variate association of family planning self efficacy with key family planning outcomes in the BIFS 2021

	Overall family planning self efficacy		Factor 1		Factor 2	
	Low [*n* (%)]	High [*n* (%)]	Low [*n* (%)]	High [*n* (%)]	Low [*n* (%)]	High [*n* (%)]
Spousal Communication (*n* = 12,786)						
No	3204 (66.1)	3409 (42.9)	2197 (69.3)	4416 (45.9)	5422 (54.3)	564 (40.5)
Yes	1645 (33.9)	4528 (57.1)	974 (30.7)	5199 (54.1)	4556 (45.7)	829 (59.5)
*p*-value	<0.0001		<0.0001		<0.0001	
Fertility intention (current/last (*n* = 11,531)						
Wanted to get pregnant	3577 (72.9)	5998 (74.3)	2293 (70.9)	7282 (74.7)	7532 (74.5)	1059 (74.4)
Wanted to wait	525 (10.7)	935 (11.6)	354 (10.9)	1106 (11.3)	1112 (11.0)	140 (9.8)
Did not want more	806 (16.4)	1140 (14.1)	583 (18.1)	1363 (13.9)	1464 (14.5)	224 (15.7)
*p*-value	0.001		<0.0001		0.2	
Contraceptive ever use (*n* = 13,411)						
No	3360 (67.0)	4739 (56.4)	2247 (68.5)	5852 (57.7)	6452 (61.8)	787 (53.3)
Yes	1654 (32.9)	3658 (43.6)	1031 (31.4)	4281 (42.2)	3989 (38.2)	689 (46.7)
*p*-value	<0.0001		<0.0001		<0.0001	
Contraceptive ever use by Type (*n* = 13,375)						
No	3360 (67.2)	4739 (56.6)	2247 (68.8)	5852 (57.9)	6452 (61.9)	787 (53.4)
Traditional Contraception	843 (16.9)	1578 (18.8)	541 (16.6)	1880 (18.6)	1899 (18.2)	299 (20.3)
Reversible Modern Contraception	793 (15.9)	2062 (24.6)	477 (14.6)	2378 (23.5)	2066 (19.8)	388 (26.3)
*p*-value	<0.0001		<0.0001		<0.0001	
Contraceptive (ever) use by type (*n* = 13,375)						
No	4523 (90.5)	7260 (86.6)	2971 (91.0)	8812 (87.2)	9266 (88.9)	1270 (86.2)
IUD	71 (1.4)	123 (1.5)	40 (1.2)	154 (1.5)	145 (1.4)	21 (1.4)
Condoms	166 (3.3)	435 (5.2)	96 (2.9)	505 (5.0)	441 (4.2)	75 (5.1)
Pills	140 (2.8)	304 (3.6)	89 (2.7)	355 (3.5)	316 (3.0)	56 (3.8)
Others	96 (1.9)	257 (3.1)	69 (2.1)	284 (2.8)	249 (2.4)	52 (3.5)
*p*-value	<0.0001		<0.0001		0.01	
Current Use of contraception (*n* = 5312)						
No	796 (48.1)	1691 (46.2)	492 (47.7)	1995 (46.6)	1843 (46.2)	344 (49.9)
Yes	858 (51.9)	1967 (53.8)	539 (52.3)	2286 (53.4)	2146 (53.8)	345 (50.1)
*p*-value	0.1		0.5		0.07	

BIFS, Bihar Integrated Family Planning Study; IUD, intrauterine devices

Multivariable analyses demonstrated associations of FP Self Efficacy with spousal communication [AOR = 2.3 (95% CI: 2.2,2.5)], contraception use [AOR = 1.4 (95% CI: 1.3,1.5)], traditional contraception [AOR = 1.2 (95% CI: 1.1,1.4)], modern contraception use [AOR = 1.6 (95% CI: 1.4,1.7)], use of condoms [AOR = 1.3 (95% CI: 1.1,1.6)], pills [AOR = 1.2 (95%: 1.02,1.5)], and with wanting to get pregnant [AOR = 1.1 (95% CI: 1.01,1.2)] (Table 5). Higher SE to discuss FP was associated with spousal communication [AOR = 2.4 (95% CI: 2.2,2.6)], traditional contraception [AOR = 1.2 (95% CI: 1.1,1.4)] and modern contraception use [AOR = 1.6 (95% CI: 1.4,1.8)], condom use [AOR = 1.4 (95% CI: 1.1,1.8)] and wanting to get pregnant [AOR = 1.2 (95% CI: 1.1,1.4)]. Higher SE to use FP was associated with spousal communication [AOR = 1.7 (95% CI: 1.5,1.9)], traditional contraception use [AOR = 1.2 (95% CI: 1.1,1.4)] and modern contraception use [AOR = 1.4 (95% CI: 1.2,1.6)].

**Table 5:** Multivariate association of family planning self efficacy with key family planning outcomes [presented as adjusted odds ratios (AORs) along with 95% confidence intervals] in the BIFS 2021

	Spousal communication (Ref No) (*n* = 12,786)	Contraceptive use (ever) (Ref No) (*n* = 13,411)	Contraceptive use (Ref No)		Contraceptive use (ever) by type (Ref No) (*n* = 13,375)				Fertility intention (Ref Did not want more) (*n* = 12,981)		Current contraceptive use (Ref: No) (*n* = 5312)
			Traditional	Reversible modern	IUCDs Use	Condom Use	Pill Use	Others	Wanted to get pregnant	Wanted to wait	
FP Self Efficacy (full)											
Low	1.00	1.00	1.00	1.00	1.00	1.00	1.00	1.00	1.00	1.00	1.00
High	2.35*(2.18, 2.54)	1.39*(1.28,1.49)	1.24*(1.12,1.36)	1.58*(1.43,1.75)	0.96 (0.71,1.30)	1.35*(1.12,1.63)	1.25*(1.02,1.54)	1.62*(1.28,2.06)	1.13*(1.01,1.25)	1.13 (0.97,1.31)	1.04 (0.92,1.17)
FP Self Efficacy Factor 1											
Low	1.00	1.00	1.00	1.00	1.00	1.00	1.00	1.00	1.00	1.00	1.00
High	2.37*(2.17, 2.59)	1.40*(1.28,1.53)	**1.24 (1.12,1.39)**	**1.63 (1.45,1.83)**	1.15 (0.81,1.65)	1.43*(1.14, 1.79)	1.26 (0.98,1.60)	1.36*(1.04,1.78)	1.25*(1.11,1.40)	1.12 (0.95,1.32)	1.007(0.87,1.16)
FP Self Efficacy Factor 1											
Low	1.00	1.00	1.00	1.00	1.00	1.00	1.00	1.00	1.00	1.00	1.00
High	1.66*(1.47,1.86)	1.34*(1.19,1.50)	1.25*(1.08,1.45)	1.43*(1.24,1.64)	0.97 (0.61,1.55)	1.13 (0.87,1.46)	1.23 (0.91,1.65)	1.50*(1.10,2.04)	0.89 (0.75,1.04)	0.76 (0.60,0.97)	0.85 (0.72,1.004)

BIFS, Bihar Integrated Family Planning Study; FP, family planning.

All models are final adjusted regression models (logistic and multinomial as appropriate) that have adjusted for age, education, caste, religion, parity, age at marriage, and family type. All values that are statistically significant at 0.05 level of significance are made bold and have an (*) in front of them.

## 4 Discussion

This study adapted and tested a measure of reproductive agency for relevance in the Indian context and examined its association with FP outcomes. The 9-item FP Self Efficacy captured a user’s confidence in overcoming barriers related to information, discussion, negotiating use and resisting pressures. We also tested family planning self efficay sub-constructs independently and these also demonstrated high reliability and validity. FP Self Efficacy was also associated with traditional and modern contraception use, including IUCDs, condoms and pills. Women’s reports of high self-efficacy to access and discuss contraception may be attributed to FP program successes in disseminating knowledge and creating awareness for FP use. High self-efficacy to discuss contraception was also associated with condom use, a type of contraception incumbent on couple interaction. High FP Self Efficacy was also associated with women’s reports of wanting to get pregnant, demonstrating greater intentionality in fertility matters.

In contrast, only 12% of women reported high FP Self Efficacy to use contraception in the face of resistance, including going against family writ. These findings show the gap between self-efficacy to discuss vs agency to use inspite of opposition, a gap that needs to be addressed by FP programs and which may reflect external resistance from husbands and partners. In most LMICs, FP programming is disproportionately targeted towards women, despite the fact that men are most often decision-makers on contraception use [35], [36]. Engaging men through gender transformative FP programming can reshape power asymmetries within couple based FP decision-making and may have implications in enabling the design of more effective programs that recognize and respond to the barriers faced by women [37], [38]. Beyond men, items of FP Self Efficacy also elicit women’s confidence to overcome resistance from key family members (e.g. in-laws or parents) who influence women’s desires, choices and behaviors related to FP. These items are direct measures of the diverse encounters by which women exercise their FP choices, and the two factors provide shorter measures to capture this agency, and going beyond measures related to decision-making, which captures the outcomes, but not the barriers and processes of women’s exercise of their agency.

The role of women’s social network is critical in a context like India, where social systems inform family planning choices, autonomy, and opportunities for many couples [39]. This can be particularly relevant in South Asian contexts where a woman’s reproductive agency cannot often be decoupled from a web of social relationships and influences navigated in the exercise of their fertility and FP choices. Understanding the scope for agency within the roles and influence of social systems and networks can provide a richer understanding of FP choices and behaviors.

Our study also showed greater resonance of family-related barriers compared to community level barriers. While FP Self Efficacy items separated family vs community barriers, in reality, norms within families and in the community may be intertwined, and even interact with one another. Women in our study reported limited engagement in community matters, indicating that in matters of family planning community takes lower priority compared to family and immediate opinions may matter more. More research is needed to unpack the contribution of family vs community barriers, and their role in shaping family planning self efficacy in different contexts.

Measures of reproductive agency within the field of sexual and reproductive health are growing, but few have been tested in LMIC contexts. We adapted this measure through a participatory dialogue with a consortium of researchers and implementers in India across the stages of design, implementation and analysis. These efforts generated a shorter, ready-to-use measure and sub-measures feasible for large-scale evaluation/ implementation research. This has tremendous scope for program design related to the demand side as it helps in understanding the user journey and how women navigate their desire for use in the family. Additionally, our efforts led to a scale with available psychomettics, and which is much shorter in nature increasing its ease of use.

Our findings need to be viewed in light of the following limitations. Firstly, FP Self Efficacy captures women’s confidence to overcome perceived barriers, and does not include material or structural dimensions of agency. This may be considered limiting as it focuses on the perception of barriers, and not objectively reported barriers to use. Secondly, in our study, we considered family planning use and spousal communication for our measure validity but did not adequately consider the complex relationship between FP self efficacy and non-use of contraception. This is a gap as like our study, women may demonstrate FP self efficacy in their choice for non-use as they may actively be trying to get pregnant. There is increasing acknowledgment that non use of contraception may also represent women’s agency [40]; however its translation into items requires careful consideration. Thirdly, our associations in the study draw from cross-sectional data and do not infer causality between FP Self Efficacy and FP outcomes. Fourthly, the survey was conducted during the COVID-19 pandemic, and contraceptive self-efficacy, access, and use may have been influenced by disruptions caused by the pandemic and consequent lockdowns. Hence, estimates may under-represent the true magnitude of relationships. Finally, we modified and adapted an existing item pool to generate FP Self Efficacy, and did not develop our own item-pool. As a result, we may have inadvertently missed aspects of FP self-efficacy relevant in this context, though our participatory approach, expert review, and field testing minimized this risk.

In conclusion, family planning self efficacy (FP Self Efficacy) as a measure of reproductive agency was found to be reliable and valid, and associated with fertility intention, spousal communication and reversible contraception use. Efforts to capture reproductive agency through measures like family planning self efficay can provide insight into the demand side determinants of contraceptive use, and aid in designing and delivering more responsive sexual and reproductive health programs that meet the needs of local communities while ensuring progress towards gender equality.

## Author contributions

A.D.: Writing – review & editing, Project administration, Methodology, Funding acquisition, Data curation, Conceptualization. Sweta Kumari: Writing – review & editing, Project administration, Data curation. P.N.: Writing – review & editing, Project administration, Methodology, Investigation, Formal analysis, Data curation, Conceptualization. T.M.: Writing – review & editing, Supervision, Project administration, Methodology, Investigation, Data curation, Conceptualization. E.E.T.: Writing – review & editing, Validation, Project administration, Conceptualization. L.McD.: Writing – review & editing, Validation, Supervision, Investigation, Funding acquisition, Formal analysis. N.B.: Writing – review & editing, Writing – original draft, Validation, Methodology, Formal analysis, Conceptualization. A.R.: Writing – review & editing, Supervision, Methodology, Investigation, Funding acquisition, Formal analysis, Conceptualization. Kalysha Closson: Writing – review & editing, Methodology, Formal analysis. A.S.: Writing – review & editing, Validation, Formal analysis.

## Declaration of Competing Interest

The authors declare no competing interests.

## Footnotes

1 The Family Planning Measurement Learning and Evaluation (FPMLE) consortium comprised a number of Indian and India-based researchers, program experts and implementers.
