# Supplementary material for: Validation of a family planning self-efficacy measure with married women in Bihar, India: Findings from the Bihar Integrated Family Planning Survey
Source: Contracept X. 2024 Nov 13;6:100113. doi: 10.1016/j.conx.2024.100113 (PMC11648775; doi:10.1016/j.conx.2024.100113)
Supplement: Supplementary Table 1 — Supplementary material [file mmc1.docx]

**SUPPLEMENTARY TABLES**

**Supplementary Table 1: Correlation between items of the Family Planning Self Efficacy in Bihar Integrated Family Planning Survey (BIFS) 2021**

|  | obtain_info | wait_line | discuss_children | discuss_method_husb | discuss_method_friend | use_w/o_discuss | use_husband_no | use_mil_no | use_parents_no | convince_husband_condom | convince_husband_male_sterilization | continue_fp_community_knowledge | use_neighbor_criticize |
| --- | --- | --- | --- | --- | --- | --- | --- | --- | --- | --- | --- | --- | --- |
| obtain_info | 1.00 |  |  |  |  |  |  |  |  |  |  |  |  |
| wait_line | 0.69*** | 1.00 |  |  |  |  |  |  |  |  |  |  |  |
| discuss_children | 0.54*** | 0.49*** | 1.00 |  |  |  |  |  |  |  |  |  |  |
| discuss_method_husb | 0.51*** | 0.47*** | 0.74*** | 1.00 |  |  |  |  |  |  |  |  |  |
| discuss_method_friend | 0.48*** | 0.44*** | 0.53*** | 0.60*** | 1.00 |  |  |  |  |  |  |  |  |
| use_w/o_discuss | 0.12*** | 0.15*** | 0.09*** | 0.13*** | 0.18*** | 1.00 |  |  |  |  |  |  |  |
| use_husband_no | 0.11*** | 0.14*** | 0.10*** | 0.13*** | 0.16*** | 0.68*** | 1.00 |  |  |  |  |  |  |
| use_mil_no | 0.20*** | 0.22*** | 0.21*** | 0.23*** | 0.26*** | 0.38*** | 0.52*** | 1.00 |  |  |  |  |  |
| use_parents_no | 0.22*** | 0.24*** | 0.24*** | 0.25*** | 0.28*** | 0.33*** | 0.45*** | 0.79*** | 1.00 |  |  |  |  |
| convince_husband_condom | 0.23*** | 0.24*** | 0.26*** | 0.27*** | 0.25*** | 0.22*** | 0.25*** | 0.28*** | 0.31*** | 1.00 |  |  |  |
| convince_husband_male_sterilization | 0.16*** | 0.18*** | 0.16*** | 0.18*** | 0.15*** | 0.29*** | 0.30*** | 0.26*** | 0.28*** | 0.55*** | 1.00 |  |  |
| continue_fp_community_knowledge | 0.39*** | 0.36*** | 0.38*** | 0.40*** | 0.42*** | 0.11*** | 0.15*** | 0.33*** | 0.38*** | 0.29*** | 0.25*** | 1.00 |  |
| use_neighbor_criticize | 0.39*** | 0.37*** | 0.40*** | 0.42*** | 0.43*** | 0.10*** | 0.14*** | 0.33*** | 0.37*** | 0.29*** | 0.24*** | 0.88*** | 1.00 |

**Supplementary Table 2: Association of Sociodemographic Covariates with Family Planning Self Efficacy in the BIFS 2021**

|  | FP Self Efficacy | | Factor 1 | | Factor 2 | |
| --- | --- | --- | --- | --- | --- | --- |
|  | High | p-value | High | p-value | High | p-value |
| Age |  |  |  |  |  |  |
| *<25* | 3081 (61.5) | **<.0001** | 3797 (75.8) | **<.0001** | 539 (11.9) | **.1** |
| *25-35* | 3512 (64.4) |  | 4188 (76.7) |  | 624 (12.9) |  |
| *35-49* | 1991 (58.0) |  | 2407 (70.1) |  | 342 (11.4) |  |
| Education |  |  |  |  |  |  |
| *<5 years* | 3540 (54.8) | **<.0001** | 4396 (68.0) | **<.0001** | 641 (10.9) | **<.0001** |
| *5-9 years* | 2165 (65.5) |  | 2620 (79.3) |  | 357 (12.2) |  |
| *10-12 years* | 1960 (67.2) |  | 2324 (79.7) |  | 342 (13.3) |  |
| *13+ years* | 919 (75.3) |  | 1052 (86.2) |  | 165 (16.3) |  |
| Wealth tertiles |  |  |  |  |  |  |
| *Poor* | 2541 (57.3) | **<.0001** | 3180 (71.7) | **<.0001** | 453 (11.3) | .05 |
| *Middle Income* | 2904 (62.5) |  | 3485 (74.9) |  | 503 (12.1) |  |
| *Rich* | 3139 (65.2) |  | 3727 (77.4) |  | 549 (13.1) |  |
| Caste/tribe status |  |  |  |  |  |  |
| *Scheduled Castes (SC)* | 1575 (60.2) | **.001** | 1907 (72.8) | **.001** | 286 (12.2) | .8 |
| *Scheduled Tribes (STs)* | 55 (46.6) |  | 74 (62.7) |  | 11 (10.0) |  |
| *Other Backward Classes (OBC)* | 5421 (62.4) |  | 6548 (75.3) |  | 935 (12.1) |  |
| *General* | 1533 (61.9) |  | 1863 (75.3) |  | 273 (12.6) |  |
| Religion |  |  |  |  |  |  |
| *Hindu* | 7325 (63.2) | **<.0001** | 8826 (76.2) | **<.0001** | 1301 (12.7) | **<.0001** |
| *Non-Hindu* | 1259 (54.3) |  | 1566 (67.6) |  | 204 (9.6) |  |
| Family type |  |  |  |  |  |  |
| *Nuclear* | 3396 (60.0) | **.001** | 4093 (72.4) | **<.0001** | 625 (12.5) | .4 |
| *Non-nuclear* | 5188 (62.9) |  | 6299 (76.2) |  | 880 (11.9) |  |
| Number of children ever born |  |  |  |  |  |  |
| *No child* | 782 (57.4) | **<.0001** | 977 (71.7) | **<.0001** | 132 (10.7) | .06 |
| *1* | 1828 (63.7) |  | 2229 (77.6) |  | 312 (12.2) |  |
| *2* | 2271 (65.8) |  | 2709 (78.5) |  | 406 (13.4) |  |
| *3+* | 3703 (59.5) |  | 4477 (71.9) |  | 655 (11.8) |  |
| Age at marriage |  |  |  |  |  |  |
| ≥18 years | 4353 (64.9) | **<.0001** | 5206 (77.7) | **<.0001** | 715 (12.0) | .6 |
| <18 years | 4231 (58.8) |  | 5186 (72.0) |  | 790 (12.3) |  |

**Supplementary Table 3: Association of Family Planning Self Efficacy (FPSE) with other Women’s Empowerment Constructs (Construct Validity) in the BIFS 2021**

|  | FP Self Efficacy | | Factor 1 | | Factor 2 | |
| --- | --- | --- | --- | --- | --- | --- |
|  | **Low** | **High** | **Low** | **High** | **Low** | **High** |
| Household Decision-making | | | | | | |
| Low | 1684 (32.6) | 1836 (21.7) | 1191 (35.2) | 2329 (22.8) | 2823 (26.6) | 392 (26.67) |
| Middle | 2962 (57.4) | 5693 (67.5) | 1804 (53.4) | 6851 (67.0) | 6809 (64.1) | 887 (60.3) |
| High | 517 (10.0) | 909 (10.8) | 386 (11.4) | 1040 (10.2) | 989 (9.3) | 191 (12.9) |
| p-value | <.0001 | | <.0001 | | <.0001 | |
|  |  |  |  |  |  |  |
| Mobility | | | | | | |
| Restricted | 3574 (67.2) | 4796 (55.9) | 2373 (67.6) | 5997(57.7) | 6771(62.4) | 863 (57.3) |
| Unrestricted | 1743 (32.8) | 3788 (44.1) | 1136 (32.4) | 4395 (42.3) | 4085 (37.6) | 642 (42.7) |
| p-value | <.0001 | | <.0001 | | <.0001 | |
|  |  |  |  |  |  |  |
| Who makes the final decision? (sub-sample, n=2536)* | | | | | | |
| Self | 200 (37.0) | 978 (49.0) | 123 (37.7) | 1055 (47.7) | 754 (42.4) | 188 (51.4) |
| Husband | 280 (51.8) | 879 (44.0) | 163 (50.0) | 996 (45.1) | 874 (49.2) | 158 (43.2) |
| NA | 60 (11.1) | 139 (6.9) | 40 (12.3) | 159 (7.2) | 150 (8.4) | 20 (5.5) |
| p-value | <.0001 | | <.0001 | | 0.004 | |

* If you and your husband disagree about using a any methods of family planning, who makes the final decision? (asked to those who said yes to contraceptive communication in last 12 months)
